# Doped Carbon Quantum Dots Reinforced Hydrogels for Sustained Delivery of Molecular Cargo

**DOI:** 10.3390/jfb14030166

**Published:** 2023-03-20

**Authors:** Shweta Kanungo, Neeta Gupta, Reena Rawat, Bhawana Jain, Aruna Solanki, Ashutosh Panday, P. Das, S. Ganguly

**Affiliations:** 1 Department of Engineering Science and Humanities, Indore Institute of Science and Technology, Indore 452001, Madhya Pradesh, India; 2 Department of Chemistry, Govt. E. Raghavendra Rao P. G. Science College, Bilaspur 495001, Chhattisgarh, India; 3 Department of Chemistry, Echelon Institute of Technology, Faridabad 121101, Haryana, India; 4 Department of Chemistry, Govt. V.Y.T. PG. Autonomous College, Durg 491001, Chhattisgarh, India; bhawanajain123@gmail.com; 5 Department of Chemistry, JNS Govt PG College Shujalpur, Affiliated to Vikram University Ujjain (M.P.), Dist Shajapur 465333, Madhya Pradesh, India; 6 Department of Physics, Dr. C.V. Raman University, Kota, Bilaspur 495113, Chhattisgarh, India; 7 Bar-Ilan Institute for Nanotechnology and Advanced Materials, Ramat Gan 5290002, Israel

**Keywords:** hydrogel, biomedicine, carbon quantum dots, colloidal stability, sustained drug delivery, present market

## Abstract

Hydrogels have emerged as important soft materials with numerous applications in fields including biomedicine, biomimetic smart materials, and electrochemistry. Because of their outstanding photo-physical properties and prolonged colloidal stability, the serendipitous findings of carbon quantum dots (CQDs) have introduced a new topic of investigation for materials scientists. CQDs confined polymeric hydrogel nanocomposites have emerged as novel materials with integrated properties of the individual constituents, resulting in vital uses in the realm of soft nanomaterials. Immobilizing CQDs within hydrogels has been shown to be a smart tactic for preventing the aggregation-caused quenching effect and also for manipulating the characteristics of hydrogels and introducing new properties. The combination of these two very different types of materials results in not only structural diversity but also significant improvements in many property aspects, leading to novel multifunctional materials. This review covers the synthesis of doped CQDs, different fabrication techniques for nanostructured materials made of CQDs and polymers, as well as their applications in sustained drug delivery. Finally, a brief overview of the present market and future perspectives are discussed.

## 1. Introduction

In recent years, hydrogels have attracted a significant amount of attention as a result of their numerous applications in biological and pharmacological fields. Hydrogels are three-dimensional polymer networks that are hydrophilic and crosslinked, and they have the ability to absorb significant volumes of water or biological fluid without being dissolved [1,2,3,4]. The existence of hydrophilic functionalities in the polymeric network, including –OH, –CONH, –CONH_2_, and –SO_3_H, contributes to their susceptibility to absorbing water [5]. The volume of water absorbed is greatly influenced by the hydrophilic/hydrophobic proportion in the polymeric chain, the kinds of crosslinker used, the density of crosslinking, and other factors. Furthermore, their swelling characteristics are affected by the swelling medium’s physicochemical properties, including pH, ionic strength, solvent, preparation techniques, solvent composition, and temperature. The term “hydrogel” was first was coined in 1894 when it was explained as an inorganic salt colloidal gel [6,7]. Poly(HEMA) hydrogel networks were first reported for use in contact lenses by Wichterle and Lim [8]. Afterwards, intelligent and responsive hydrogels were fruitfully used in horticulture and agriculture as actuators, separation procedures for water blocking tapes, removal and separation of heavy metals, as well as for sustained drug delivery, protein separation, tissue engineering, etc. [9,10,11,12,13,14,15]. Because of their high water content and soft consistency, which is comparable to that of natural tissues, they find widespread applications in a variety of products, including contact lenses, biosensors membranes, linings for artificial hearts, materials for artificial skin, and devices for delivery of drugs [16,17].

Nanomaterials, such as quantum dots (QDs), which exhibit intriguing electrical and optical properties and have applications in a wide variety of domains, such as biological imaging, photovoltaic systems, energy storage, and others [18,19,20,21]. However, the majority of QDs are made up of harmful heavy metals, including cadmium, lead, and mercury, which restricts their practical applicability. Carbon quantum dots (CQDs) are a relatively recent addition to the carbonaceous family. They are recognized as distinct, quasi-spherical, clustered regions of many carbon atoms with sizes of less than 10 nm. Due to their outstanding water solubility, excellent biocompatibility, chemical inertness, and photoluminescent features, CQDs have recently garnered significant attention [22,23]. Because of their exceptional features, CQDs hold great potential for a wide range of applications, including cell imaging, clinical diagnosis, solar conversion, sensors, drug administration, catalysis, and photovoltaic devices [11,22,24,25]. CQDs are one of the most advanced and superior nanomaterials, so extensive research into their incorporation into polymer matrices is important. The reinforcing action of CQDs in polymer matrices has been the subject of discussion and reported by a number of different researchers [26,27]. Therefore, QDs confined hydrogels is a hot topic among materials scientists and biomedical researchers [28,29,30,31]. High tensile strength (161.4 kPa) and stretchability (842.1% elongation at break) were demonstrated by hydrogels when CQDs were present into the system. This remarkable expansion can be accounted for by the physisorption of the gel matrix onto the CQDs surface, which takes place via secondary contacts (H-bonding or polar–polar interactions) [32]. In addition to its superstretchability, researchers have discovered that certain types of hydrogels have the ability to self-heal. Certain hydrogels also emit photoluminescence. Chen and his fellow researchers were able to create an enhanced CQDs-containing hydrogel that possessed remarkable self-healing characteristics [33]. In this study, we set out to explore a wide variety of confined quantum dots (CQDs) hydrogels and their applications in the sustained administration of therapeutic molecules. 

## 2. What Are CQDs? 

**Figure 1:**
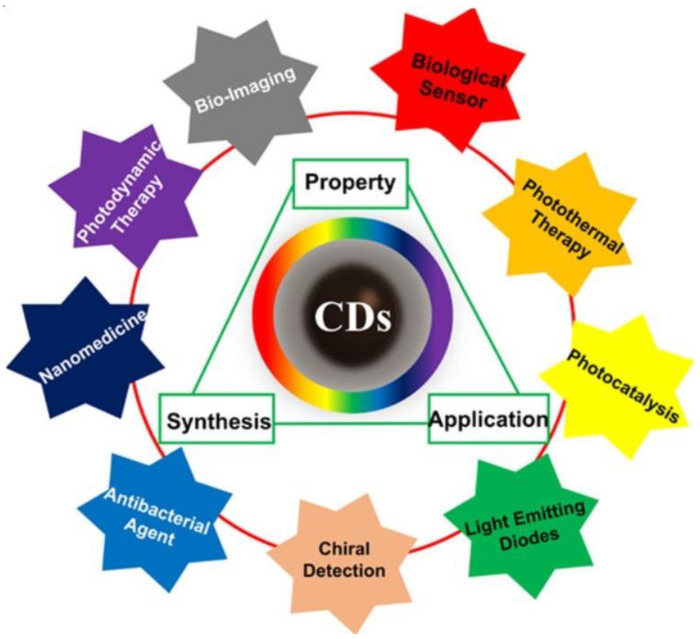
The triangular representation of the corrections among synthesis, property, and application of CQDs with wide application ranges [43].

Among the various carbon allotropes, CQDs (Figure 1) have become one of the star representatives among the various carbon allotropes due to their ease of preparation and intriguing characteristics, including excellent biocompatibility, water stability, good chemical and photostability, and low toxicity. CQDs are carbon-based nanomaterials with dimensions less than 10 nm [34,35,36]. The first time they were acquired was in 2004 during the process of purifying SWCNTs using preparative electrophoresis [37]. After that, in 2006, they were obtained through the laser ablation of graphite powder and cement [38]. CQDs are envisioned as a possible substitute for metal-based semiconductor QDs due to their remarkable qualities, such as low toxicity, water dispersibility, good optical properties, bio-compatibility, and eco-friendliness [39,40,41,42]. Figure 1 shows the different types of applications of CQDs [43].

## 3. General Synthetic Techniques of Doped CQDs

**Figure 2:**
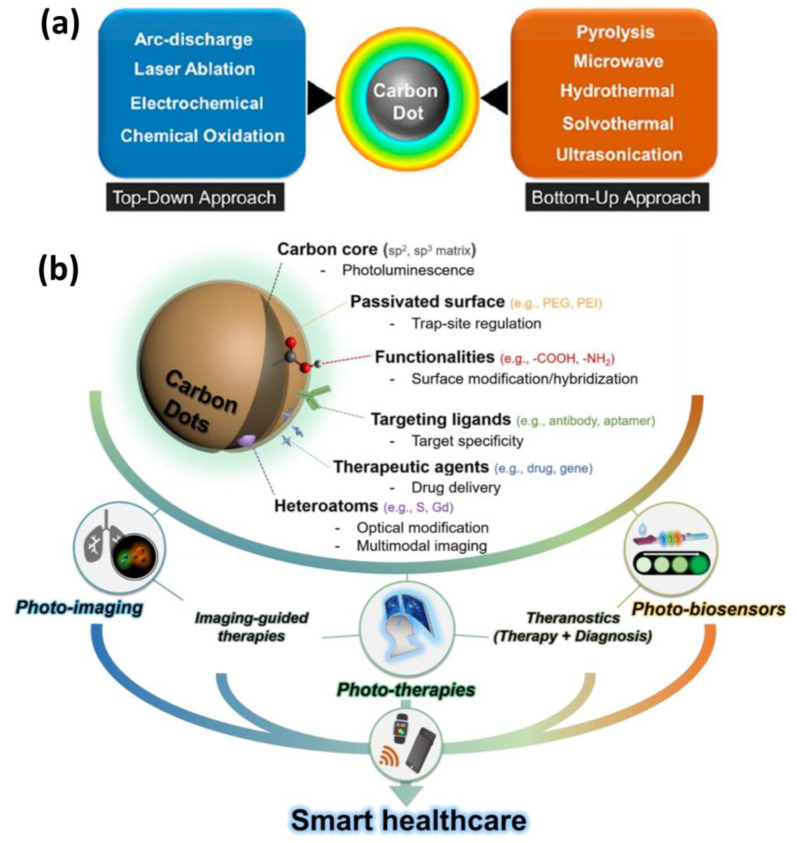
(**a**) Schematic representation of top-down and bottom-up methods for CQD synthesis (**b**) their surface modifications, target specificity, drug delivery, multimodal imaging and applications in smart healthcare [53,54].

Top-down and bottom-up methodologies can be used to categorize CQDs fabrication methods [22,44]. Top-down approaches use physicochemical methods to break down macromolecules or large molecules into smaller fragments, whereas bottom-up approaches use polymerization or carbonization of small molecules to convert them to nanomaterials through the use of chemical reaction [22,45]. The thermal decomposition of the precursors can be accomplished through the utilization of a variety of processes, such as reflux under acidic or basic conditions, hydrothermal treatment, chemical oxidation, and ultrasound- or microwave-assisted synthesis [22]. Compared to top-down techniques, which call for pre-existing aromatic structures, bottom-up approaches allow for the synthesis of CDs using nearly any organic material susceptible to thermal degradation [46,47]. The top-down synthesis approaches involve arc discharge methods, laser ablation, plasma treatment, electrochemistry, etc., whereas bottom-up fabrication methods include hydrothermal, pyrolytic process, microwave-assisted, combustion, etc. [22,34,41,46,48]. The upsides of the top-down method include large-scale production, abundant raw materials, and simple operation. Furthermore, this method of synthesis typically contains oxygen functionalities at the edges, which improves their solubility and surface passivation. The top-down method, on the other hand, has a number of drawbacks, including low yield, the use of specialized equipment, the risk of damaging the aromatic carbon structure, and the use of a method of chemical cutting that is not selective. Because of these challenges, this technique does not provide for particularly precise control over the shape and size distribution of the nanoparticles [49]. The bottom-up strategy, on the other hand, offers numerous opportunities to control the morphology, size, and thus properties of the QDs [50]. In these synthetic experiments, hydro/solvochemical decomposition is used to prepare CQDs from small organic molecules, amino acids, and other naturally occurring starting materials (such as sugars). It is important to note that among the several carbon sources that are accessible for CQD bottom-up synthesis, citric acid and amino acids are common choices as CQD precursors [51]. On the other hand, these processes frequently need the use of a complex synthesis method and a certain category of organic precursors, both of which might be challenging to obtain. Huge efforts have been made in recent years to develop novel synthesis procedures for CQDs and GQDs, and the aforementioned drawbacks have gradually been resolved through intricate designs [52]. The typical synthesis procedures of CQDs in healthcare applications are depicted schematically in Figure 2.

## 4. Different Doping Strategies of CQDs

Doping is a common method used to tune the luminescent characteristics of photoluminescent materials. To tune the attributes of CQDs, a number of doping techniques using a large number of elements, including N, S, and P, have been documented [55,56,57]. Doping can be categorized as single-doped or co-doped according to the number of hetero atoms injected into the CQDs [58]. The incorporation of heteroatoms (such as N, P, and S) into the CD structure greatly enhances the luminescent properties of these nanomaterials, increasing the fluorescent quantum yield and changing the absorbance and emission bands towards a blue or red shift [36]. Because of these factors, during the course of the last ten years, a significant amount of scientific effort has been spent on the production of dopants and strategies for doping. The dopant most frequently used to enhance the photoluminescent characteristics of CQDs is nitrogen. Injecting electrons into CQDs modifies their internal electrical environment, enhances their fluorescent properties, and induces outstanding catalytic activity, low cytotoxicity, and exceptional cell permeability. Incorporating nitrogen into the carbon framework has been successfully accomplished through both the top-down and bottom-up methodologies [59]. In comparison to the number of studies on N doping, CQDs with a single sulphur (S) atom have received less attention. However, S-doped CQDs have attracted a lot of attention in recent years due to the fact that sulphur atoms could provide energy or emissive trap states for photostimulated electron capture, thereby altering the electronic structure of CQDs. There have been reports of S-doped CQDs being synthesized by means of pyrolysis, ultrasonication, microwaves, and hydrothermal/solvothermal processes [35,60]. P doping can act as an n-type impurity in nanodots. The size of the P atom is greater than that of the carbon atom. As a result, it is capable of behaving in a similar fashion as an n-type donor, which results in the generation of defects in the carbon cluster as well as a change in the electrical and optical characteristics of CQDs [61]. Boron (B), like N and S, can be effectively doped into CQDs via covalent doping. Doping CQDs with B may be a viable method for modifying their electrical and optical properties. B-doped CQDs have excellent water solubility and exhibit brilliant fluorescence in both the solid and liquid states. Because of this, they are utilized as agents for cell imaging as well as convertor elements for use in light-emitting devices [62,63]. Dopants such as Si, Zn, Cu, Fe, Gd, and Eu, in addition to the above discussed elements, have been used to adjust the electrochemical, biological, and optical properties of CQDs in order to make them more suitable for a wide range of applications [64,65]. 

### 4.1. In-Situ Anchoring in Polymer Matrices 

**Figure 3:**
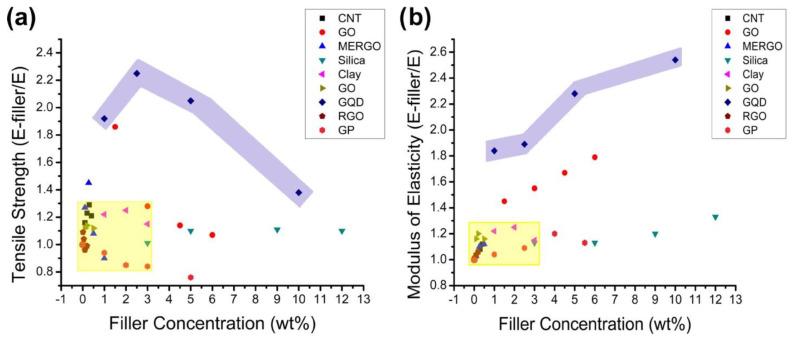
(**a**) The ultimate tensile strength of various nanoparticles was compared with that of neat epoxy, demonstrating that GQDs offered good enhancement. (**b**) Comparing the moduli of elasticity of composites of various nanoparticles to those of pure epoxy showed that GQDs stiffened the epoxy relative to all the other nanoparticles. In both graphs, the GQDs are showcased in the purple shaded region [69].

Nanoparticles have established a path for tailoring polymer matrix characteristics [66]. Due to their propensity for agglomeration and inappropriate distribution across the polymer matrix, high surface energy is a significant challenge for polymer chemists. Nevertheless, their surface polarity and surface functionalities make them suitable for nano-inclusion in polymeric matrices. Due to insufficient stress dissipation, lack of homogeneity in reinforcement, and poor thermal stability, the service life of polymer composites is occasionally compromised. Several studies have used nanoclay, graphene platelets, silica, graphene oxide (GO), nanodiamonds, CNTs, and other fillers in polymer matrices [67,68,69,70,71,72]. In this sense, CQDs play an essential role in resolving the aforementioned shortcomings. Such characteristics of CQDs are merely a reflection of their surface properties and the polar anchoring process between the functional groups of the polymer and CQDs. Additionally, polymers play the role of stabilizing high-surface energy nanoparticles. CQDs can also promote physisorption-assisted polymer chain attachment on their surfaces. Surface functional groups are extremely susceptible to bonding with the polymer’s pendant polar functional groups. Figure 3 compares the advantages of GQDs over various nanofillers. By comparing the influence of GQDs and other nanoparticles on epoxy matrices, including nanoclay, CNTs, silica, GO, and graphene platelets, it was confirmed that GQDs had the most significant effect [69]. As can be seen in Figure 3a, it was discovered that GQDs were able to improve the characteristics of epoxy by 2.3 times, but 90% of the other nanoparticles could only achieve an improvement of 1.3 times. This method was able to obtain a higher degree of homogeneity in the dispersion of nanoparticles than other methods. A parallel pattern of findings was noticed while examining the elasticity of the modulus. As can be seen in Figure 3b, the addition of GQDs to epoxy produced a tensile strength that was 2.5 times greater than that achieved by any of the other nanomaterials. Many different types of functional groups, such as carboxyl, amino, and aldehyde groups, can be found on the surface of CQDs. Because of its structural feature, CQDs are highly soluble in water and can interact with gelators in a variety of noncovalent ways, making them a promising class of building blocks for the development of hydrogels. Furthermore, the surface functionalities of CQDs could react with the gelator or be further modified as needed, resulting in chemically linked network systems. These advantages allow for the potential of combining CQDs and hydrogels, which is beneficial to both components and results in a win–win situation [73]. Immobilizing CDs within hydrogels has been shown to be a smart tactic for preventing the aggregation-caused quenching effect when they are in aggregated form [74]. The incorporation of CDs into hydrogels results in the enhancement of a number of features, including luminescence, chirality, stimulus responsiveness, self-healing, and mechanical and biological properties [75,76]. The combination of these two entirely different kinds of materials not only results in a diversity of structures but also an enormous improvement in many other properties, which leads to the creation of new materials with multiple functions.

### 4.2. Categories of Hydrogel

**Figure 4:**
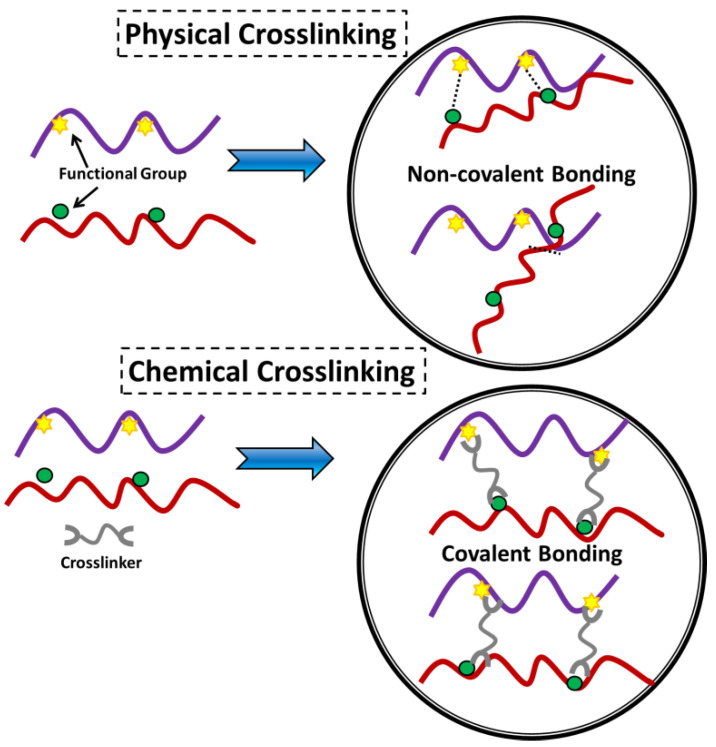
Illustration of physical and chemical crosslinking displaying the type of connection within the material [82].

In general, hydrogels can be categorized according to a variety of attributes, such as the nature of the pendant groups (cationic, anionic, or neutral), the method of fabrication (homo or copolymer), the structural and mechanical properties (amorphous, semi-crystalline, hydrogen-bonded, supramolecular, and hydrocollodial), and the ability to respond to environmental stimuli. Other characteristics that can be used to classify hydrogels affect the ability of the hydrogel to absorb water (pH, ionic strength, temperature, electromagnetic radiation, etc.) [40,77,78,79,80,81]. The chemical or physical nature of the crosslink junctions is another factor that can be used to classify hydrogels into two further categories. These types of hydrogels are referred to as (i) physically crosslinked hydrogels and (ii) chemically crosslinked hydrogels, as depicted in Figure 4 [82].

## 5. Physically Crosslinked Hydrogels

Physically crosslinked hydrogels are also known as reversible gels. In reversible gels, the network that holds the gel together is composed of either polymer chain entanglements or physical interactions, including ionic interactions, hydrogen bonding, or hydrophobic interactions. Due to the physical interactions that exist between distinct polymer chains, dissolution is inhibited in a physically crosslinked gel [17,83]. These interactions are reversible and susceptible to disruption by alterations in environmental variables or the application of stress.

## 6. Chemically Crosslinked Hydrogels

**Figure 5:**
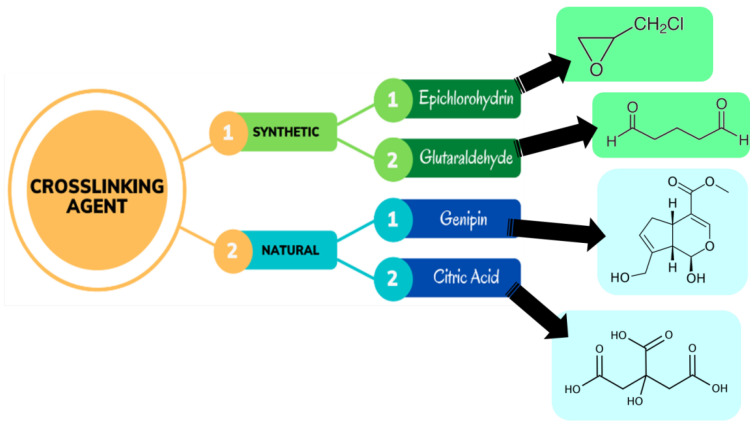
Different crosslinking agents used in the synthesis of hydrogels [85].

The development of covalent bond between molecular chains to construct a three-dimensional network that connects molecules is known as chemical crosslinking. Chemically crosslinked hydrogels are also referred to as ‘permanent gels’. This is because the network of the hydrogel comprises permanent junctions. The equilibrium swelling ratio of these different forms of hydrogels is determined by the interactions between the polymer and water along with the density of the crosslinking [17,84]. As a result of covalent bonding, the crosslinker is linked to at least two polymer backbones in this example. The crosslinking agent used in the synthesis of hydrogels can be derived from either natural or synthetic sources, as shown in Figure 5 [85]. There has been widespread usage of crosslinkers such as glutaraldehyde, epichlorohydrin, bisacrylamide, etc. to make crosslinked hydrogel networks composed of a wide range of synthetic and natural polymers.

## 7. Release Mechanism

**Figure 6:**
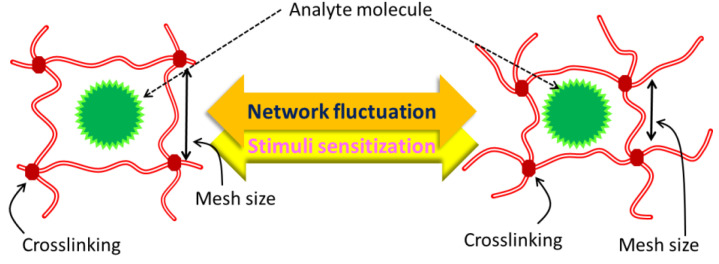
Size of hydrogel mesh in expanded and contracted states, schematically.

The chemical or physical crosslinking of individual polymer chains results in hydrogels, which are polymeric networks that absorb enormous amounts of water but are insoluble in aqueous solutions. Hydrogels may be used in a variety of applications. Natural polymer hydrogels have various benefits, including intrinsic biocompatibility, biodegradability, and physiologically identifiable moieties that promote cellular functions, but they may not provide enough mechanical qualities and may contain infections or elicit immune/inflammatory responses. However, these intrinsic bioactive qualities are not present in synthetic hydrogels. Fortunately, the structures of synthetic polymers are often well-defined, allowing for modifications to produce specialized degradability and usefulness. Traditional medication delivery methods sometimes call for either large doses or repeated administration to induce a therapeutic effect, which can decrease overall efficacy, patient compliance, and even cause toxicity. When the mesh size is comparable to the solute size, as illustrated in Figure 6, it is theoretically impossible for the solute to diffuse inside the hydrogel matrix. A gel’s mesh size responds to a number of variables, including (a) its degree of crosslinking, (b) the chemical structure of its component monomers, and (c) environmental stimuli including temperature, pH, and ionic strength. The mechanical strength, biodegradability, and molecular diffusion rate of hydrogels are all significantly affected by the mesh size.

**Figure 7:**
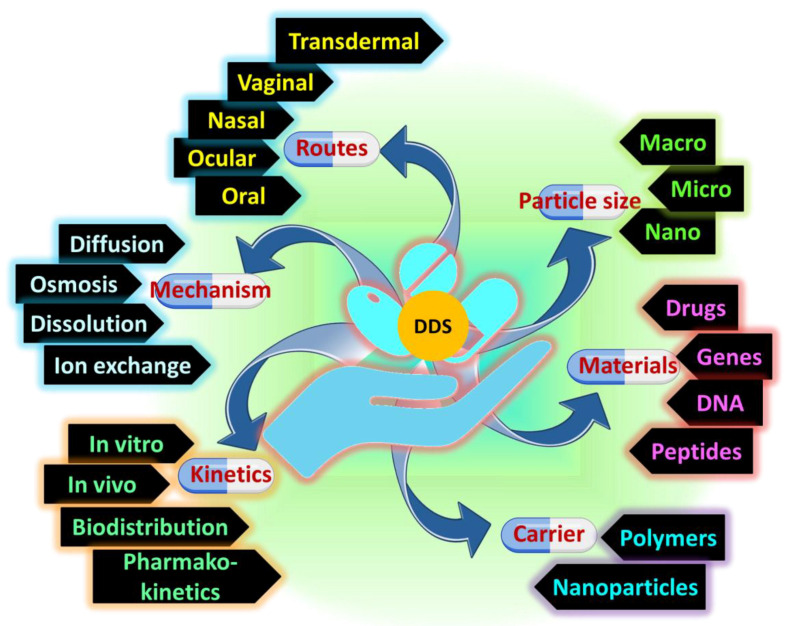
The advancement of drug delivery from fundamental research to applications.

Biodegradability, crosslinking type, source, ionic charge, manufacturing process physical qualities, and response nature are only a few of the criteria used to categorize hydrogels. Figure 7 provides a comprehensive taxonomy of hydrogels. When it comes to physical gels, the crosslinking process is, well, physical. Hydrophobic association, chain aggregation, crystallization, polymer chain complexion, and hydrogen bonding are typical physical mechanisms that accomplish crosslinking. In contrast, chemical hydrogels are made using a chemical process, namely, chemical covalent crosslinking (either concurrently or post-polymerization). Conformational modifications allow physical hydrogels to be reversed, but configurational changes in chemical hydrogels make them permanent and irreversible. Dual-network hydrogels, generated by the electrostatic interaction of physically and chemically crosslinked hydrogels, are another kind of hydrogel. Because of their high liquid absorption capacity throughout a broad pH range and greater sensitivity to changes in pH than chemical hydrogels, these have lately been used to counteract the limitations of employing either kind of hydrogel alone. It was recently revealed elsewhere that another dual-network made of graphene–polymer composites have exceptional mechanical characteristics and a self-healing ability [86,87].

Access to the human body by hydrogels is highly dependent on their macroscopic architecture. Hydrogels are extremely malleable and may be shaped into nearly any three-dimensional form. Convective drug transport is possible if micropores are present, which has a major impact on the material’s overall physical qualities (such as its deformability). The water in the hydrogel is encased in a crosslinked polymeric network that extends across several nanometers in size. The mesh size refers to the average distance between nodes in a given network. How medications disperse within the hydrogel network is critically dependent on the mesh size. Finally, medicines and polymer chains may engage in a wide range of chemical reactions at the molecular and atomic scales. Multiple locations for binding interactions with pharmaceuticals can be pre-designed into the polymer chains utilizing a variety of physical and chemical approaches.

Hydrogels, as we have seen, provide a special set of benefits for usage in drug administration because of their unusual composition. Hydrogels are able to absorb a lot of water (>90% by weight) because of their hydrophilicity. Thus, hydrogels differ greatly from hydrophobic polymers in the ways through which molecules are released. It is possible to forecast the release of an active substance from a hydrogel device over time using either simple or complex models. The following groups of models can be identified by their focus on the rate-limiting stage in controlled release: diffusion-controlled, swelling-controlled, and chemically controlled.

The most frequently applicable mechanism for characterizing drug release from hydrogels is diffusion-controlled release. Diffusion-controlled release can be modeled using either a constant or variable diffusion coefficient according to Fick’s law of diffusion. The study of solute transport in hydrogels has several potential uses. Hydrogels have several uses in the biomedical and fermentation industries, as well as in chromatography, biosensing, transport of bioactive compounds to the body, prosthetics, and other separation processes [88]. The unique capacity of hydrogels to limit a solute’s diffusive mobility is used in each of these applications. The factors influencing solute diffusion within hydrogels, as well as the methods through which they affect diffusion, are thus crucial to comprehend. Thus, several mathematical formulas have been created to attempt to represent solute transport in hydrogels. The goal of this article is to analyze and contrast the most popular models using data from the literature to determine how well they predict outcomes. Muhr and Blanshard conducted such an analysis back in the day, but since then, many different models have been offered [89]. The water-filled areas defined by the polymer chains are where solute transport in hydrogels takes place. The solute’s mobility can be affected by anything that diminishes the available space between the molecules. Among these are the relative sizes of the solute and the openings between the polymer chains, the mobility of the polymer chains, and the presence of charged groups on the polymer that may bind the solute molecule. The mobility of the polymer chains plays a significant role in regulating solute transport inside the hydrogel. Electrode materials made of CoO quantum dots on 3D graphene hydrogels were produced by Wang et al. and they required no binder [90]. It was reported by Wu et al. that a class of polysaccharide-based hybrid nanogels could integrate the functional building blocks for optical pH sensing, cancer cell imaging, and controlled drug release into a single nanoparticle system [91]. This has the potential to offer broad opportunities for combined diagnosis and therapy [92,93]. The hybrid nanogel was made by in-situ immobilization of CdSe quantum dots (QDs) within the interior of a dual pH- and temperature-responsive hydroxypropylcellulose-poly(acrylic acid) (HPC-PAA) semi-interpenetrating polymer network. This allowed for the nanogel to respond to both changes in pH and temperature. Mouse melanoma B16F10 cells could have their intracellular regions illuminated by the hybrid nanogel since it weas able to penetrate cellular barriers and enter the intracellular space. The hybrid nanogel showed exceptional stability as a drug carrier, which not only allowed for high drug-loading capacity of the model anticancer agent TMZ, but it also offered the possibility of pH-regulated drug administration. According to the findings of investigations on the kinetics of drug release, multifunctional nanogels exhibit sustained drug release characteristics, and release of the drug may be initiated by the pH-responsive volume phase transition of the gel. Emerging drug delivery techniques based on nanotechnology allow for the selective killing of cancer cells while causing minimal harm to healthy tissue. One study discussed the synthesis, characterization, and evaluation of a mitomycin C (MMC)-encapsulated chitosan (CS)-based nanocarrier system comprised of Mn:ZnS quantum dots (QDs) [94]. To specifically target cancer cells for therapeutic delivery, a nanocarrier containing the chemical doxorubicin (DOX) was created. This was accomplished by first synthesizing harmless Fe_3_O_4_ nanoparticles (NPs) using the co-precipitation technique [95]. The NPs were then given a hydrophilic and biocompatible polyethylene glycol (PEG) surface functionalization to make them more stable. Graphene quantum dots (GQDs) were used to embellish the Fe_3_O_4_@PEG, which allowed it to take on new optical features and boost its drug-loading capability. When tested, the generated nanocarriers (Fe_3_O_4_@PEG@GQD) demonstrated excellent superparamagnetic capabilities in addition to minimal toxicity, a hydrodynamic diameter of 129 nm, and a drug-loading percentage of 27%. Studies of drug release showed a pH-dependent profile, with greater release rates at acidic pH (5.0) than at the physiological pH range (7.4). Fe_3_O_4_@PEG@GQD-DOX inhibited the growth of human breast cancer cells as effectively as free medicine. In order to examine in vitro sunitinib (STB) administration using a luminescence spectrometer, a polymer dot-modified histidine-functionalized graphene quantum dots carrier, PD@His.GQD, was created [96]. A chitosan-based hybrid nanogel (Rh 100 nm) was described, containing CdSe QDs (3.2–3.8 nm) in-situ immobilized in the chitosan-poly(methacrylic acid) (chitosan-PMAA) semi-IPN network [97]. CdSe QDs were developed as an optical identifier for use in biosensing and cellular imaging. The pH-regulated physical characteristics, toxicity, and functions of hybrid nanogels made using the covalent crosslinking technique and the physical association method were studied and compared.

Swelling-controlled release happens when diffusion of the medication occurs more quickly than swelling of the hydrogel [98,99]. The modeling of this process often requires the use of shifting boundary conditions, in which molecules are allowed to escape at the border between the rubbery and glassy phases of inflated hydrogels [100]. At room temperature and body temperature, swelling-controlled release systems are generally polymers that have a glassy consistency. The glassy polymer first pushes back against the water’s attempt to penetrate it, but it gradually allows water to enter the free volume near the surface. The glassy polymer that is located on the surface relaxes into a structure that is more water-friendly, and as a result, it expands. This makes it possible for even more water to penetrate the structure, and one may frequently notice a moving front dividing a swelling outer layer from a dry inner core. In most cases, swelling is followed by a shift from the glassy state to the rubbery state. If the medication is held captive inside the glassy state, it will be released as the polymer expands, and if it is able to diffuse through the loosened matrix more quickly than water can enter, then the release process is regulated by swelling. The dynamics of swelling are frequently complicated and a wide range of temporal release patterns can be seen under controlled conditions. It is possible that, given the right circumstances, swelling, the breakup of polymer chains, and drug release may all happen at the same time, adding still another layer of complexity.

## 8. Fabrication Techniques of Carbon Dots Reinforced Hydrogels

**Figure 8:**
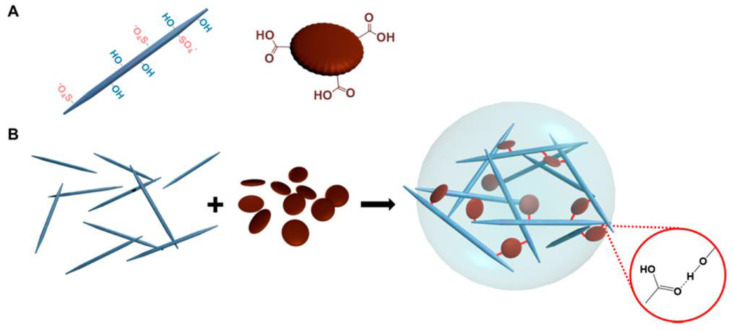
Diagram of CNC-GQD hydrogel. (**A**) Hydrogel building blocks: CNC with surface hydroxyl and half-ester sulphate groups (**left**) and GQD with edge carboxylic groups (**right**). (**B**) CNC-GQD suspension hydrogels. The inset depicts the hydrogen connection between GQD carboxyl and CNC hydroxyl groups. Reproduced with permission from ref. [103].

New technologies that combine established ones have led to significant strides in materials science. Here, hydrogels stand out as a potential material for a wide range of biomedical and biological applications thanks to their ability to mimic human soft tissues via their 3D matrix, flexibility, and high water content. Innovative combinations of nanomaterials, in particular graphene quantum dots (GQDs), offer the potential of bestowing higher functionality to the nanocomposite hydrogel, which might have applications in a wide range of industries and help make the most of the distinctive features and functionalities of hydrogels. As a result, the hydrogel’s mechanical strength, rheological characteristics, etc., are all improved thanks to a synergistic effect. When coupled with other materials to form composites, hybrid hydrogels exhibit a broad variety of mechanical, chemical, thermal, and electrical behaviors, which contribute to their widespread use. N-GQDs, or nitrogen-doped graphene quantum dots, were created to further investigate and broaden their use in the biomedical industry [101]. First, at concentrations between 10 and 100 g/L, we tested the isolated N-GQDs for their hemocompatibility and cytotoxicity. Nanocomposite hydrogels are based on the premise that they provide a microenvironment similar to that of real tissue, one that is conducive to nutrition exchange via a porous structure and cell growth. A hydrogel incorporating nitrogen-doped carbon dots (NCDs) has been manufactured and crosslinked to create a hybrid injectable and biodegradable hydrogel based on oxidized alginate/gelatin [102]. In vitro biodegradation and swelling behavior experiments showed that adding up to 0.06% NCDs reduced the swelling ratio and weight loss of the hydrogel. The biocompatibility of the composite hydrogel was confirmed by MTT test in MG-63 cells. Degradation and interaction within cells and the hydrogel were significantly influenced by the N-doped graphene quantum dots. A hydrogel formed from CNCs and graphene quantum dots (GQDs) has been reported (Figure 8) in terms of its characteristics and versatility [103]. Even though CNCs and GQDs are both negatively charged, they demonstrate that hydrogen bonding and hydrophobic interactions may overcome the electrostatic repulsion between these nanoparticles to produce a physically crosslinked hydrogel with controllable mechanical characteristics.

**Figure 9:**
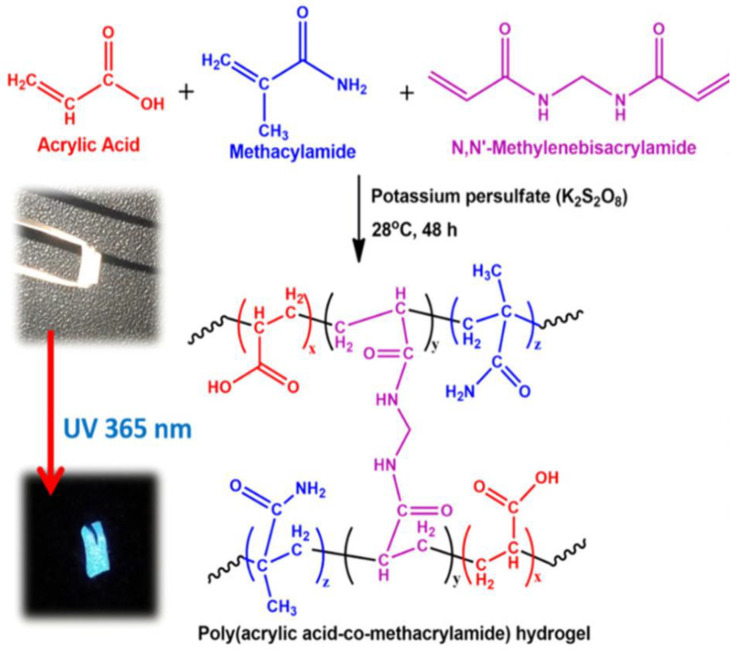
Hydrogel synthesis reaction mechanism that makes sense (inset: hybrid hydrogel images in visible and 365 nm UV light). Reproduced with permission from ref. [11].

One-pot green hydrothermal treatment was used to successfully create a hydrogel (NCDs/CNF-gel) composed of fluorescent nitrogen-doped carbon dots and cellulose nanofibrils [104]. The authors investigated the mechanisms responsible for NCD self-assembly in the hydrogel network and for the enhancement of the hydrogel’s compressive strength. The blue-green fluorescence emission of the NCDs/CNF-gel was both acid- and alkaline-sensitive. Fabrication of fluorescent carbon dots from a green tea precursor has been reported. Using these tea carbon dots, a hydrogel film was successfully created that exhibited superior physico-mechanical capabilities than a chitosan hydrogel film crosslinked only with glycerol [105]. Another study revealed the use of alginate-derived nitrogen-doped CDs as a drug carrier and toughening agent for hydrogels [11]. In this work, a thermal coupling technique using microwave irradiation was devised for the production of multipurpose CDs. Alginate, a highly abundant polysaccharide, was employed to form the core of the spherical CDs particles and surface decorating was performed with urea as the N-doping ligand. Composite hydrogels (Figure 9) have been shown to be mechanically resilient, with properties including high stretchability (1200% elongation at break), low permanent set, controllable water retention, and thixotropic behavior under dynamic stress. Evaluation of the crosslinked structure by void computation revealed a steady compactness of the connection with higher CD concentration.

**Figure 10:**
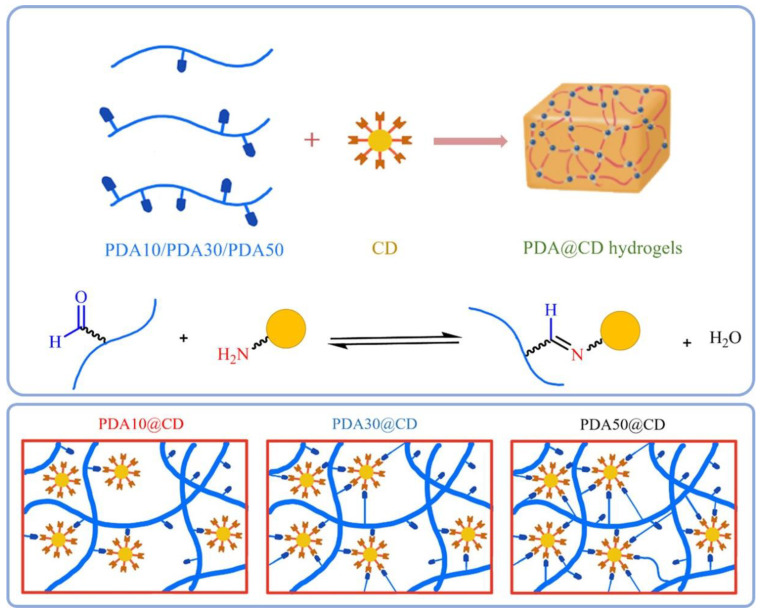
Schematic illustrations of dynamic covalent bond-formed PDA@CD hydrogels with different degrees of oxidation and molecular weights. Reproduced with permission from ref. [107].

In response to the need for less invasive surgical treatments, Wang et al. created a unique carbon dots/hydroxyapatite/PVA (CDs/HA/PVA) dual-network (DN) hydrogel scaffold with good fluorescence and biocompatible qualities [106]. The composite hydrogel was made using a two-step process that involved first chemical crosslinking and then physical crosslinking. They also demonstrated that the chemically crosslinked SN hydrogel had pores of varying sizes (50–100 µm) and a porous structure. NC hydrogels were developed by Lu et al. by crosslinking polydextran aldehyde (PDA) polymers by imine bond synthesis utilizing amine-functionalized carbon dots (CDs) [107]. It was found that among the three PDA@CD hydrogels (Figure 10) tested, the PDA50@CD hydrogel had the densest structure and maximum mechanical strength due to its higher oxidation degree compared to the other two hydrogels.

**Figure 11:**
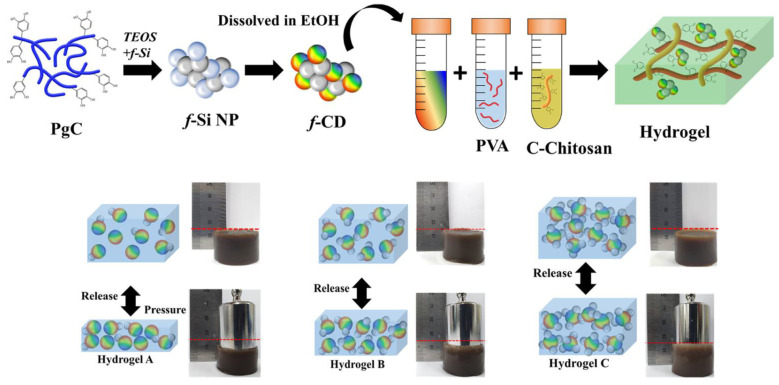
Diagrammatic representation of CD synthesis and hydrogel production. Reproduced with permission from ref. [108].

A hydrogel for use in pressure and vibration sensors was developed by Ryplida et al. by combining hydrophobic carbon dot nanoparticles (f-CD) with polyvinyl alcohol and catechol-conjugated chitosan [108]. A hydrogel made of polyvinyl alcohol (PVA) and carboxymethyl chitosan (C-chitosan) was prepared using the synthesized hybrid carbon-silica fluorescent nanoparticles (f-CD), as shown in Figure 11. Details about the hydrogel’s electrical response to mechanical forces, including pressure, tension, and vibration, were revealed throughout the design process by manipulating the hydrogel’s f-CD affinity. Tuning the f-Si ratio affected the hydrogel matrix due to the hydrophobicity of the hydrogel being dependent on the quantity of fluorinated silane (f-Si) loaded in the f-CD. Simply put, the mechanical strength and water swelling ratio of hydrophobic hydrogels are superior to those of hydrophilic gels. This is because the hydrophobic interactions between nanoparticles and matrices impart a stiff shape.

**Figure 12:**
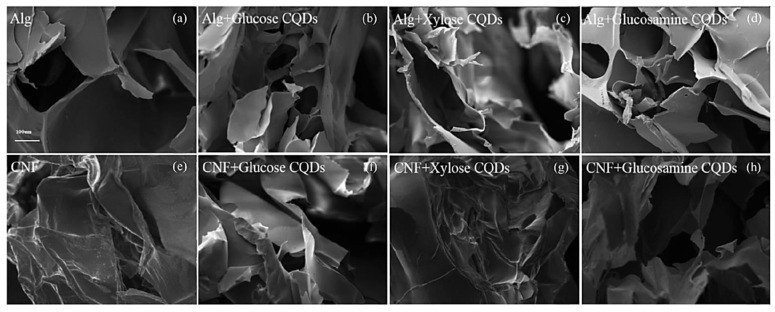
Scanning electron micrographs of (**a**) ALg hydrogel, (**b**) ALg + CQDs-1 hydrogel, (**c**) ALg + CQDs-2 hydrogel, (**d**) ALg + CQDs-3 hydrogel, and (**e**–**h**) CQDs-CNF hydrogel (Mag = 150×). Reproduced with permission from ref. [110].

It has been reported that soft polypyrrole (PPy) hydrogels can be easily fabricated with in-situ-doped sulfonated graphene quantum dots using a simple synthetic process (sGQD) [109]. Electrostatic interactions between the sulfonic acid groups surrounding the graphene quantum dots (GQD) and the nitrogen groups of PPy lead to the chains of PPy forming a three-dimensional network that may be gelated into a hydrogel. In order to increase performance, it has been shown that sGQD may generate transport channels to speed up the diffusion of solvated ions during the charging and discharging processes. By protonating the nitrogen groups, the sGQD molecule is able to engage with more than one PPy chain, building a 3D structure through electrostatic interactions due to the sulfonic acid groups. The resulting tangled PPy chains can function as transport channels, allowing for rapid solvated ion diffusion throughout the charge and discharge cycle. Hydrogels made from alginate (ALg) and cellulose nanofibers (CNF) were functionalized with fluorescent biomass carbon dots to create a fully biodegradable fluorescent hydrogel (CQDs) [110]. Biomass CQDs served a dual purpose in the composite hydrogels. First, the CQDs added strong fluorescent characteristics to the hydrogels. Second, the CQDs helped in crosslinking the numerous oxygen-containing groups or amino groups on the surface with alginate or cellulose nanofibers, thereby improving the mechanical characteristics of the hydrogels. Figure 12 displays scanning electron micrographs (SEM) of the CQDs-ALg and CQDs-CNF hydrogels. Observation of the alginate hydrogel revealed a porous material with a pore size of 100–200 nm and a loose lamellar structure characterized by smooth and thick cell walls. The CNF hydrogel displayed the same lamellar structures. Furthermore, this study demonstrated that the microstructures of composite hydrogels are little affected by the inclusion of CQDs.

**Figure 13:**
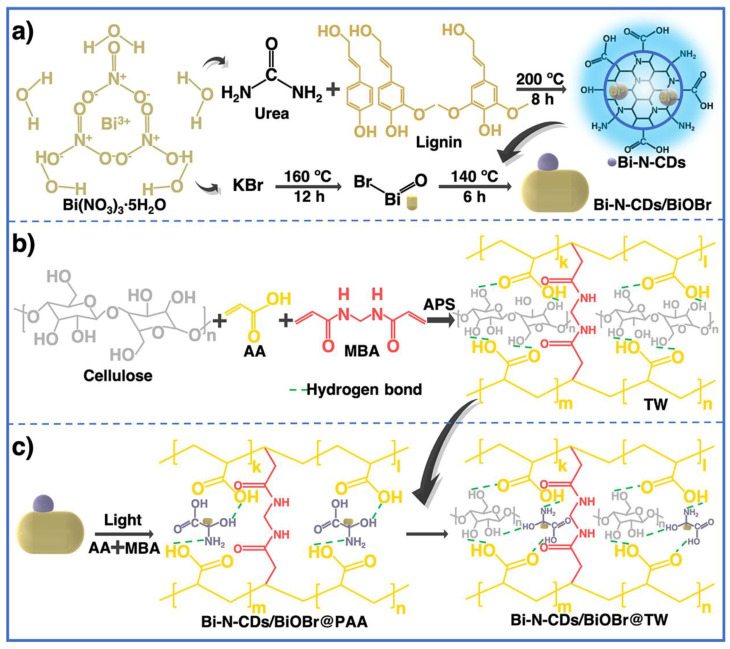
(**a**) Synthesis of Bi-N-CDs and Bi-N-CDs/BiOBr, (**b**) formation mechanism of transparent wood (TW), (**c**) formation mechanism of Bi-N-CDs/BiOBr@PAA and its interface connection with TW. Reproduced with permission from ref. [113].

A tridimensional hydrogel consisting of S,N-GQD nano-islands encased in NC fibrils was reported to be easily fabricated by Ruiz-Palomero et al. This method not only improved the luminescence (PL) characteristics of GQDs but also prevented their aggregation via stacking of nanosheets [111]. The suggested hydrogel had exceptional sensing capabilities towards 2,4,5-trichlorophenol due to its novel optical characteristics. Red wine and water samples were used to verify the accuracy of the procedure and show that it was suitable for rapid screening of TCP. Carbon dots (CDs) and magnesium fluorohydroxyapatite (MFA) were utilized to functionalize carbon nanotubes (f-CNTs) to create a unique f-CNT-CD-MFA hybrid, as previously described. Because of its superior physicochemical and adsorption capabilities, the hybrid was introduced into the alginate matrix [112]. Good interfacial attachment between the filler and matrix was achieved by the application of sonication waves, which also contributed to an increase in the heat resistance of pure alginate as a result of this rapid and environmentally friendly approach to preparation. For the purpose of removing dye, Li et al. manufactured a core–shell-constructed wood hydrogel using a PAA-crosslinked cellulose fiber framework (core matrix layer) and Bi-N-CDs/BiOBr-initiated PAA hydrogel (shell functional layer), where delignified wood served as a reinforcing backbone and Bi-N-CDs/BiOBr acted as an initiator and photocatalyst (Figure 13) [113]. This method provides a path towards reducing dye pollution by providing a means of producing wood hydrogels that are exceptionally stretchable, transparent, dye removal efficient, and recyclable.

## 9. Diffusion and Physico-Mechanical Characteristics of Carbon Dots-Based Hydrogels

**Figure 14:**
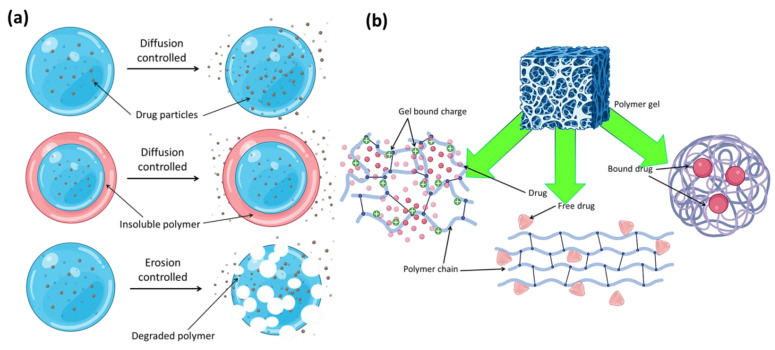
(**a**) Polymer–drug conjugates and different types of release mechanism. (**b**) Different types of probable drug–drug conjugates in polymer gel networks.

If a medicine is metabolized too quickly and excreted from the body too soon after delivery, this method of drug release can be very helpful. By controlling the rate at which a medication is released, sustained release maintains a consistent concentration of the drug in the blood or targeted tissue. Sustained drug release can be achieved, according to some research, by delaying the moment at which drug molecules enter the aqueous environment. Tuning the breakdown rate of a carrier or the diffusion rate of drug molecules through an insoluble polymer matrix or shell allows for the detection of this inhibition (Figure 14a). As a result of their high water content, most hydrogels allow for a relatively fast release of pharmaceuticals from the gel matrix over the course of hours or days, especially in the case of hydrophilic medications. The time required for this release profile is far less than that possible with microspheres or macroscopic devices based on more hydrophobic polymers. Many methods have attempted to slow the pace at which drugs are released from hydrogels. One way to classify these methods is by whether or not they raise the diffusive barrier to release of the drug from the gel network or improve drug–matrix correlations. In order to increase the coupling between a loaded drug and the hydrogel matrix and hence the length of drug release, both physical and chemical techniques can be applied, as seen schematically in Figure 14b. Stronger contacts between the gel and target medication have often been achieved by making use of charge interactions between ionic polymers and charged medicines. The efficacy of phosphate-functionalized polymers stems from their inherent multivalent anionic charge. Carbohydrate-based polymers often include both anionic and cationic functional groups, and both can significantly affect the release of an oppositely charged medication. The effects of sodium ibuprofen’s association with cationic celluloses and cationic guar gums on the substance’s aqueous dispersions and crosslinked hydrogels were studied by Rodríguez et al. [114]. Polymer molecular structure was evaluated using four different types of cationic polymers, two types of cationic hydroxyethyl celluloses, and two types of cationic guar gums. Because the medication and sodium dodecylsulfate may both be interested in binding to the polymer, the latter’s presence was also taken into account to see how it would affect the former.

To control drug release, thermosensitive switches are commonly used in combination with surface-specific alterations to create a reduced permeability “film” layer at the hydrogel surface. The surface permeability of hydrogels and consequently their release kinetics may be controlled by temperature by grafting thermosensitive PNIPAM polymers onto their surface in this way [115]. Using the inverse emulsion polymerization process, a nano drug carrier consisting of a thermo-responsive nanohydrogel was developed and used to transport doxorubicin [116]. Using a straightforward bottom-up method involving the pyrolysis of citric acid, GQDs were made and subsequently incorporated into the nanohydrogel to enhance the gel’s LCST and anticancer capabilities. Intravenous injection of B16F10 cell lines into the tail veins of C57BL/6 mice resulted in the development of a murine melanoma model. To begin exploring the potential of doxorubicin (DOX)-loaded nanohydrogels for the treatment of lung metastasis, we first evaluated their effects on body weight, organ weight, and number of lung metastatic sites. The possible toxicity and immunogenicity of the DOX-loaded nanohydrogels associated with prognosis were also assessed by hematological and cytokine tests. As antibiotic resistance among pathogens rises, so does the urgency with which we must investigate novel wound healing agents. New wound dressings need to be developed to facilitate wound cleaning, adequate wound moisture, and rapid epithelization. In this research, graphene quantum dots (GQDs) were infused into bacterial cellulose (BC) for possible use in wound healing [117]. Human fibroblasts migrated significantly after being exposed to GQDs-BC hydrogels, as shown by an in vitro healing investigation. Additionally, following 72 h of exposure to GQDs-BC, the fibroblast gene expression levels of endothelial nitric oxide synthase, vascular endothelial growth factor A, matrix metallopeptidase 9, and Vimentin were considerably elevated, supporting angiogenesis. Making composite hydrogels that are both self-healing and mechanically strong is a formidable task. In this work, we showed that a composite hydrogel made of graphene oxide and poly(ethylene glycol) may be used to solve this challenge by striking the right balance between components (acrylic acid–acrylamide) [118]. With a selectivity of 90–95%, the hydrogel was able to remove organic cationic dyes from polluted water (methylene blue vs. rhodamine B). Dye adsorption on this composite hydrogel appeared to follow a pseudo-second-order model, as shown by the kinetics analysis. In a recent study, Kharlampieva et al. reported the successful fabrication of spin-assisted layer-by-layer hybrid materials, including quantum dots, immobilized in a responsive photoluminescent way [119]. Poly(allylamine hydrochloride) (PAH) and poly(sodium 4-styrenesulfonate) (PSS) are examples of strongly interacting polyelectrolytes that can be used to confine thioglycolic acid-stabilized CdTe nanoparticles, and a poly(methacrylic acid) (PMAA) hydrogel matrix demonstrated an elastomeric network with pH-responsive properties. Wound care management via in vitro and in vivo investigations revealed the antibacterial action of pH-responsive TA/KA composite hydrogel crosslinked with GQDs (TA/KA-GQDs) utilizing citric acid as a crosslinker [120]. In addition, the produced samples were evaluated in an in vivo wound healing model with a wound size of 1.5 cm^2^. Using the produced samples for wound healing resulted in complete closure of the wound regions after 16 days of therapy. To demonstrate that keratinocytes could proliferate on biocompatible and biocomposited TA/KA-GOQDs, a histological examination was performed using MT and HE staining.

## 10. Sustained Release Application

When a medicine is designed for sustained release, it can be released gradually over time, allowing it to have an effect over a larger patient population.

Hydrogels are able to influence the microenvironment of a tissue because of their porous and hydrated molecular structure [121]. Chemical or physical crosslinking is used to create hydrogel networks. In physiological settings, a three-dimensional hydrogel network made from highly hydrated polymers materials can absorb water from the surrounding environment and expand without dissolving [122]. Therapeutic effectiveness may be compromised in traditional drug delivery systems due to the medication’s rapid initial release followed by a less-than-optimal concentration in the body’s fluids and cells. Hydrogel nanocomposite beads based on carboxymethylcellulose (CMC) were prepared using a novel physical crosslinker, copper acetate. The given approach has the potential to garner significant interest in the field of controlled release of pharmaceuticals due to the mildness, simplicity, and generation of tiny and uniformly shaped CMC-based hydrogel nanocomposite beads. Hydrogel beads were prepared with a model medication called naproxen (NPX) already inside [123]. The produced beads’ potential as an oral drug-delivery device was demonstrated by drug release studies conducted under settings mimicking the in vivo procedure. The beads were tested in a medium with pH values of 1.2, 6.8, and 7.4 over the course of numerous intervals. Additionally, raising the concentration of GQD reduced the NPX release percentage. Whilst one would anticipate that as the GQD concentration is raised, the swelling rate of nanocomposite beads will decrease, the opposite was seen. Havanur et al. reported the synthesis and characterization of a novel drug carrier made from poly(N,N-diethyl acrylamide) (PDEA) and graphene quantum dots (GQDs) [124]. The inclusion of GQDs enhanced the already impressive properties of PDEA, a stimuli-responsive, macroporous polymer that can adapt to changes in environmental temperature. Using free radical polymerization, PDEA hydrogels and GQDs were produced in this study. Doxorubicin (DOX) is an anthracycline used in cancer treatment and research into its release behavior has revealed that hydrogel performance is affected by hydrogel composition, time, and environmental temperature. Hydrogels containing GQDs and PDEA showed promising cytotoxicity data, lending credence to their use as smart drug carriers. Nanoparticles engineered for therapeutic uses often have a combination of bioinert and physiologically relevant functional groups on their surface. Hume et al. developed a PEG hydrogel system in which the mesh size was adjustable to be either larger or smaller than the precisely regulated nanoparticle diameter. The release of nanoparticles from hydrogels may then be studied in relation to the mesh size of the hydrogels and the nanoparticles themselves [125]. Three-dimensional poly(ethylene glycol) hydrogels of varied mesh sizes were used to enclose functionalized gold nanoparticles or quantum dots. Particle release from the hydrogel matrix was shown to be affected by three factors: nanoparticle size, surface chemistry, and hydrogel mesh size. As predicted, nanoparticle release slowed down with increasing particle size.

## 11. Present Market and Future Perspectives

The last two decades have seen a worldwide surge of interest in nanotechnology, leading to a steady stream of new inventions in fields as disparate as bioengineering, biochemistry, pharmacology, fabric, and food science. According to the literature, the synthetic polymers used in the construction of nanotechnological materials make them prone to degradation into unfavorable compounds and render them incapable of interacting with cells. As a result, this is a key issue with nanomaterials that raises concerns in the biomedical sector. However, the medical community is very interested in particle systems such as metallic nanoparticles (NPs) because of their ability to stifle microbial development (bacteria, fungi, and viruses). The capacity of hydrogels to hold and release medications and provide a humid environment has piqued the interest of biomedical researchers in recent years. Therefore, one of the other routes towards increasing the efficacy of treatment systems to make them extremely successful and with reduced unpleasant side effects is the discovery and development of hydrogel-incorporated metallic NPs from natural sources. Several scientists have manufactured nanoparticles and their nanocomposites in parallel with the development of hydrogels and their eventual arrival into the commercial market during the past few decades. The safety of nanoparticle technology remains a key concern, despite the fact that nanoparticles are finding increasing use in consumer products today. Integrating them with hydrogels is one way to get around this problem and reduce the hazards to people and the planet. In addition to creating materials with unique structures, fusing these two types might improve their overall performance. Hydrogel networks serve as nanoreactors for the production of nanoparticles due to the enormous voids within them. To create nanocomposite hydrogels, polymeric hydrogels serve as a “host”, welcoming a variety of nanoparticle “guests”.

Hydrogels, with their hydrophilicity and microporous structure, appear to be a fantastic host for nanomaterials. Nanoparticles made of carbon, polymers, silica, and metals or metal oxides are only a few of the guests in hydrogel networks [41]. Most advances in the study of multifunctional materials may be attributed to the fact that scientists have been able to unite these formerly separate fields of study under one umbrella. Stimuli-responsive, catalytic, antibacterial, and wound-healing hydrogels with tunable characteristics are only some of the multifunctional nanocomposite hydrogels that have been produced and will be explored in the future. Each of these nanocomposite hydrogels possesses its own unique combination of characteristics that makes it suitable for a distinct field of biomedical research. Thus, the emergence of innovative nanocomposites with astute functionalization procedures may prove to be the most fruitful for realizing future progress. It is currently difficult to mass produce nanocomposites in an industrial setting. Recent advances in processing technologies, such as microfluidic reactors and 3D printing, present the opportunity to fabricate nanocomposite hydrogels with engineered architectures at a large scale. Researchers from a variety of disciplines have taken an interest in QDs-based hydrogels due to the unique synergistic features that are not present in single component hydrogels. QDs-based hydrogel sensing systems have mostly been studied for their ability to improve hydrogel characteristics, such as strengthening the hydrogel’s crosslinked structure and improving its mechanical and solubility. Nevertheless, there is a dearth of research into how QDs’ luminous characteristics may be altered in a hydrogel matrix. Due to its unique 3D network structure, which is chemically inert, the growing size of QDs and the diffusion rate of the reaction solution can be precisely regulated. Consequently, it will be crucial to study ways to rationally construct experiments using QDs or polymers to better regulate the development and non-aggregation of QDs in the gel matrix. Hydrogels commonly experience swelling and dehydration in real-world sensing application settings due to their propensity to absorb and then lose water. From a theoretical standpoint, hydrogel-based composites are quickly becoming the chosen alternative to replace fossil-based materials due to their outstanding material qualities. In the not-too-distant future, hydrogels will overtake other raw materials as the go-to for producing lightweight, high-strength composites for green industrialization. Eco-friendly materials for the effective design of reinforced materials, cost-cutting initiatives, increased energy efficiency, and reduced environment-related consequences are only some of the projected outcomes of this trend.

## 12. Conclusions and Future Outlook 

Attaching hydrophilic functional groups to a polymer backbone allows for the creation of a new 3D network in addition to high water absorption capacity. The outstanding tissue-mimicking behaviors that are produced as a result of the microstructural tailoring capacity of fluorescent hydrogels are impressive. Carbon dots (CD)- and polymer-based nanocomposite hydrogels have lately emerged as potential new materials with integrated properties of their constituent elements. This is because these materials combine the properties of their constituent parts. Recent years have witnessed a number of important developments in hydrogel nanocomposites formed of CQDs and polymers; these developments are addressed in this article. This article also explores the production of nanocomposite hydrogels based on CDs and polymers, as well as their prospective applications in areas such as environmental remediation, energy storage, sensing, drug delivery, and bioimaging. Using CDs to enhance the mechanical characteristics of polymer hydrogels is a somewhat unexplored area at the moment in research. Although crucial in the study of luminous nanoparticles and soft substances, research into the luminescent modulation of CDs using hydrogel networks is lacking. In the future, researchers may be able to regulate the reactive groups, topologies, and condensed structures of polymers in order to accomplish fluorescence modulation of nanocomposite hydrogels. This may allow researchers to fine-tune the various interactions that occur between CDs and polymers. Nanocomposite hydrogels will soon be able to be manufactured in a wide variety of colors, including fluorescent and phosphorescent variants. These hydrogels have the potential to be used in bioimaging, multi-responsive sensing, and tissue engineering, all of which are promising fields of application.

## Data Availability

Not applicable.
